# Small Is Beautiful: Is ctDNA Ready for Routine Implementation in Cancer Management?

**DOI:** 10.3390/cancers18132034

**Published:** 2026-06-23

**Authors:** Caroline Bailleux, Jean-Marc Ferrero, Rym Bouriga, Loic Trapani, Baharia Mograbi, Jocelyn Gal, Gérard Milano

**Affiliations:** 1 Department of Medical Oncology, Antoine Lacassagne Center, University Côte d’Azur, 33 Avenue de Valombrose, 06189 Nice, France; 2 Medical Oncologist, Park Imperial Clinic, 28 Boulevard du Tzarewitch, 06000 Nice, France; 3 Department of Pathology, Antoine Lacassagne Center, University Côte d’Azur, 33 Avenue de Valombrose, 06189 Nice, France; 4 Institute for Research on Ageing and Cancer, Nice (IRCAN), Institut Hospitalo-Universitaire (IHU) RespirERA, Fédérations Hospitalo-Universitaires (FHU) OncoAge, Centre National de la Recherche Scientifique (CNRS) 7284, Institut National de la Santé et de la Recherche Médicale (INSERM) U1081, University Côte d’Azur, 06107 Nice, France; baharia.mograbi@univ-cotedazur.fr (B.M.); gal.j@ihu-respirera.fr (J.G.); 5 Scientific Direction, Antoine Lacassagne Center, University Côte d’Azur, 33 Avenue de Valombrose, 06189 Nice, France

**Keywords:** ctDNA, tumor markers, liquid biopsies, minimal residual disease, NGS, artificial intelligence

## Abstract

Circulating tumor DNA (ctDNA) has emerged as a transformative tool in cancer diagnostics, enabling the non-invasive detection of tumor-derived DNA fragments released into the bloodstream through cellular lysis or active secretion. ctDNA measurement has demonstrated its clinical usefulness, including early cancer detection, identification of resistance mechanisms, and screening of asymptomatic individuals. In addition to prognosis, ctDNA analysis is increasingly used to guide adaptive treatment strategies by detecting minimal residual disease and tracking tumor evolution in real time. Recent advances in artificial intelligence are poised to further enhance the clinical impact of ctDNA, transforming it from a passive monitoring biomarker into a dynamic molecular sensor integrated into predictive clinical decision models. However, broad implementation of ctDNA-based assays in routine practice requires rigorous prospective validation, cross-platform standardization, and regulatory approval to unlock its full potential in precision oncology.

## Simple Summary

Circulating tumor DNA, composed of small fragments of cancerous DNA, can be detected through a simple blood test. This innovative approach offers a less invasive way to monitor cancer progression over time, detect residual disease after treatment, catch relapse earlier, and observe how tumors evolve or develop resistance to therapy. In this short review, we explore how circulating tumor DNA is measured, its current benefits in cancer care, and the challenges it still faces. We also discuss exciting new computational tools, including artificial intelligence, that could transform circulating tumor DNA from a basic monitoring method into a powerful tool for guiding personalized treatment decisions. While progress is rapid, broader clinical use of circulating tumor DNA will depend on standardizing techniques, conducting prospective validation, and establishing clear regulatory frameworks. These advances have the potential to strengthen personalized cancer management and more adaptive, effective treatment strategies.

## 1. Background

The evolution of tumor markers is a long saga that began five decades ago with the identification of tumor-released proteins, notably alpha-fetoprotein and CEA [1]. More recently, and in line with evolving clinical practice, certain tumor markers have become essential for managing patients with various types of cancer, especially those such as the estrogen receptor and human epidermal growth factor receptor 2, which are used as the sole criteria for clinical decision-making [2]. Overall, the use of tumor markers has significantly contributed to the active follow-up of treated cancers, providing easily applicable tools to evaluate therapeutic efficacy and deliver early warning signals of tumor progression [3].

**Figure 1:**
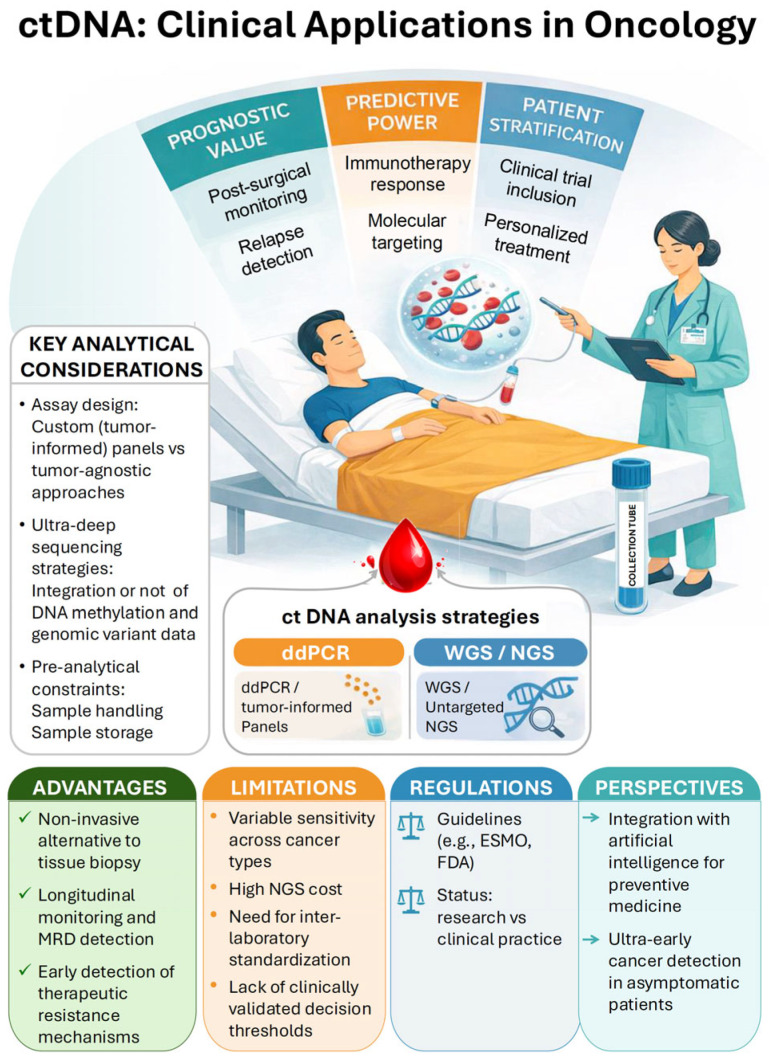
Circulating tumor DNA (ctDNA) analysis enables minimally invasive tumor profiling through blood-based liquid biopsy. ctDNA can provide prognostic information, including post-surgical monitoring and early detection of relapses through longitudinal sampling. It also has predictive value, enabling identification of molecular alterations associated with response to targeted therapies or immunotherapy. In addition, ctDNA supports patient stratification, facilitating inclusion in clinical trials and guiding personalized treatment strategies. Two main analytical strategies are used for ctDNA detection. Targeted approaches, such as droplet digital PCR (ddPCR) or tumor-informed sequencing panels, enable ultra-sensitive detection of predefined mutations and are particularly suited for minimal residual disease monitoring. In contrast, genome-wide approaches, including whole-genome sequencing (WGS) or untargeted next-generation sequencing (NGS), allow broader characterization of tumor genomic alterations and clonal evolution. Despite its clinical promise, ctDNA analysis faces several challenges, including variable sensitivity across tumor types, high sequencing costs, limited inter-laboratory standardization, and the absence of universally validated clinical decision thresholds. Ongoing regulatory efforts and integration with emerging computational approaches may further expand the clinical utility of ctDNA in precision oncology.

The last 50 years have also seen an extraordinary increase in understanding the mechanisms of carcinogenesis, summarized as the hallmarks of cancer [4], along with rapid advances in analytical methods, including the emergence of multi-omics–based analyses [5]. Liquid biopsies, as a non-invasive approach, offer a broad panel of early tumor markers from a single blood sample. This reflects the expanded knowledge of cancer biology and provides information on circulating tumor cells, exosomes, and circulating free nucleic acids [6]. Among the latter, circulating tumor DNA (ctDNA) consists of tumor DNA fragments released into the circulation through cellular lysis or active secretion by tumor cells [7] (Figure 1). ctDNA measurement has the theoretical advantage of depicting intra-patient spatial and temporal tumor heterogeneity more precisely than tumoral tissue sequencing [8].

Here, we focus on ctDNA because it represents a minimally invasive biomarker with strong potential for detecting minimal residual disease (MRD), with several examples in the monitoring of colorectal cancer and lung cancer [9]. We highlighted recent key reviews in the field and discussed major contributions illustrating the clinical relevance of ctDNA in cancer management, covering screening, diagnosis, treatment, and follow-up. Our article selection prioritized randomized multicenter studies with large participant groups. Critical analytical aspects, including the recent applications of omics methods, as well as limits and opportunities, were also objectively considered in this selective narrative synthesis dedicated to ctDNA measurement in cancer management.

## 2. ctDNA—Technical Aspects

Most ctDNA exists as double-stranded fragments approximately 150–200 bp long, with a variable low proportion of ultrashort (around 50 bp) fragments [10]. The results of ctDNA analyses depend on several factors, primarily the assay type, the amount of released cell-free DNA, and the technical and biological background [11]. Different approaches can be used to detect tumor-related mutations in ctDNA samples in clinical settings [12]. First, genomic information can be obtained through tailor-made panels, a tumor-agnostic approach [13], or by identifying common cancer driver genes that are often not captured through direct tissue sequencing [14] (Table 1). Tumor-agnostic assays that determine ctDNA content are generally useful for standard disease monitoring [15]. In contrast, ctDNA assays based on personalized, tumor-informed technology can outperform fixed-panel assays in prognostic analysis [16]. To clarify, the tumor-informed and the tumor–agnostic (or tumor-naïve) assays differ essentially in that the molecular profile of the tumor is either known (tumor-informed) or not (tumor-agnostic). It follows that tumor-informed methods necessitate sequencing of the patient’s tumor and identifying the somatic mutations specific to the tumor. The conception of a genomic personalized test follows, which precisely targets these mutations.

Overall, the latest-generation assays capable of ultra-sensitive detection can identify the presence of ctDNA with a sensitivity limit of 1 part per million (ppm), meaning the proportion of tumor-derived DNA fragments among one million DNA fragments. Specifically, Black and colleagues demonstrated that ctDNA detection below 80 ppm has clinical utility during adjuvant therapy, enabling risk stratification in lung cancer patients [17]. Similarly, in breast cancer patients, Garcia-Murillas and coworkers described an ultrasensitive WGS-based, tumor-informed ctDNA platform that can detect tumor relapse with a median lead time of 15 months over clinical evidence of relapse [18]. The notion of assay sensitivity for ctDNA measurement must be contextualized, taking into account several factors, such as the input DNA, the tumor fraction, and the pre-analytical handling [17,18].

However, a rare somatic mutation may often be difficult to detect even with ultra-deep sequencing techniques of the ctDNA sample, mainly due to the limited plasma volume collected during routine testing. Interestingly, to address these challenges, whole-genome sequencing approaches have been developed that use machine-learning guidance to analyze single-nucleotide and copy-number variants, thereby enabling significant enrichment of ctDNA signals [19]. Additionally, accurate tracking of MRD through ctDNA analysis has been achieved with an assay that combines methylation and genomic variant data [20].

Certain concrete limitations may hinder the widespread use of ctDNA analyses in everyday settings [21]. As the authors underline, these challenges involve various factors, including sample handling and storage, which are complicated by ctDNA’s very short half-life. Another potential limitation may arise from the presence of clonal hematopoiesis in blood samples. The fact is that blood samples from individuals with clonal hematopoiesis may yield false positives due to mutations in genes present in the hematopoietic clone that are misidentified as mutations in cancerous solid tumors. Therefore, further efforts are needed to establish standardized and easily interpretable ctDNA analysis results for routine use (Figure 1).

## 3. ctDNA—Clinical Correlates

The ESMO recommendations on the use of ctDNA for cancer management were updated in 2022 [8]. While recognizing ctDNA’s strong clinical potential to identify actionable mutations for targeted therapy, especially for Epidermal Growth Factor Receptor (EGFR) pathogenic mutations guiding lung cancer treatment, the expert panel concluded that ctDNA measurement still needs to demonstrate its clinical utility in several areas through three main approaches. These include the early identification of patients who are not responding to treatment, monitoring therapy to detect emerging resistance and its molecular mechanisms, and screening asymptomatic individuals for cancer. Since then, many of these issues have found concrete answers as follows.

A strong prognostic value of ctDNA testing was demonstrated in an important study focused on tumor location across the spectrum of cancer sites. It was a retrospective analysis of 834 HER2-positive metastatic breast cancer patients exploring the associations between confirmed response to T-DX and baseline characteristics. Notably, responders had tumors with high HER2 expression, lower ESR1 gene expression, and also interestingly, lower ctDNA levels [22]. A well-conducted phase I/II trial in lung cancer patients treated with anti-EGFR therapy revealed that baseline ctDNA presence significantly predicted disease outcomes (PFS, OS) [23].

Response to treatment could also be predicted early through the dynamic evolution of ctDNA (ctDNA clearance), as reported in locally advanced resectable esophageal squamous cell carcinoma [24]. On a broader scale, Valenza and coworkers conducted a systematic review of clinical trials (13 studies, 380 patients) investigating ctDNA clearance and pathologic complete response (PCR) in a neoadjuvant setting under checkpoint inhibitors [25]. More specifically, a lack of ctDNA clearance appeared as a faithful indicator of an incomplete response to treatment. The authors, however, emphasized the high heterogeneity of the data, which they attributed to variability in ctDNA assays, and thus recommended further research in this area. Importantly, the results of the PREDICT-DNA trial were recently reported [26]. It is the first multicenter prospective ctDNA trial in stage II-III HER2-positive breast cancer to establish that an ultrasensitive (detection limit to 100 parts per million) ctDNA determination after neoadjuvant treatment is an independent prognostic marker of the risk of recurrence. Additional insights came from a study of patients with early-stage NSCLC, in which ctDNA clearance after neoadjuvant therapy was associated with improved pathological response, progression-free survival (PFS), and overall survival (OS), with hazard ratios ranging from 0.26 to 0.63 [27]. Postoperative ctDNA detection indicated MRD and was correlated with a significantly higher risk of relapse. Beyond its prognostic value, ctDNA-based detection of MRD opens the possibility of adaptive therapeutic strategies, in which treatment intensity could be escalated in ctDNA-positive patients at high risk of relapses or safely de-escalated in ctDNA-negative patients. Strengthening the connection between ctDNA and tumor evolution, an adjuvant therapy that cleared ctDNA was associated with improved recurrence-free survival [27]. Interestingly, post-operative ctDNA positivity could predict recurrence up to six months before imaging [27].

On the other hand, in a longitudinal study design, Lv J and coworkers performed an unsupervised clustering analysis of EBV-associated nasopharyngeal carcinoma, identifying patients with distinct profiles of overall ctDNA evolution dynamics and different survival outcomes [28]. The PARERE trial exemplifies the discriminative power of ctDNA in a strictly controlled trial setting [29]. It was the first large, randomized study to establish the efficacy and utility of ctDNA-guided anti-EGFR monotherapy rechallenge in patients without RAS/BRAF-V600E mutations. Results from the PARERE trial showed that anti-EGFR therapy was superior to the later-line tyrosine kinase inhibitor regorafenib in overall response rate, disease response rate, and progression-free survival. Tie and colleagues advanced this concept by using ctDNA to guide treatment strategies in stage 3 colon cancer through a multicenter randomized phase II/III trial [30]. Briefly, for ctDNA-negative patients, de-escalation significantly reduced oxaliplatin use and hospitalizations. In the same vein, it was reported that a substantial clinical benefit was observed in metastatic breast cancer with an adapted targeted therapy following the detection of ESR1 mutations in metastatic breast cancer through ctDNA analysis, and this before clinical progression [31]. It must be emphasized that improvements in survival through treatment modification based on ctDNA remain context-dependent and not universally applicable.

Previously, qualitative rather than quantitative ctDNA test results have been shown to have clinical value. Notably, Turner and coworkers demonstrated the value of rapid ctDNA genotyping in selecting breast cancer patients for targeted therapies against rare HER2 and AKT1 mutations (1051 participants in a multicenter phase IIa prospective trial) [32]. Another interesting post hoc analysis examined the plasma BRAF-V600E allele fraction (AF) in patients with metastatic colorectal cancer and tissue-confirmed BRAF-V600E mutations [33]. The authors reported that among patients with high BRAF AF, survival was longer for those receiving the triple therapy (RAFi-MEKi-anti-EGFR) than for those treated with the doublet (RAFi-anti-EGFR). No survival differences were observed among patients with low BRAF AF regardless of treatment combination. These findings suggest that, despite the approval of the doublet combination by health agencies on both sides of the Atlantic, ctDNA-based analysis could identify a subset of BRAF-V600E-mutated patients who might benefit from adding a MEK inhibitor. Such evocative results warrant further investigation from well-designed prospective studies.

As complementary aspects regarding ctDNA, for patients with aggressive prostate cancer, Herberts and coworkers performed serial WGS analyses on ctDNA and compared sequencing results before and after clinical progression on androgen deprivation [34]. The authors found, through ctDNA genomic analyses, a population with highly recurrent copy-number changes, mutations, and structural rearrangements at the androgen receptor locus, suggesting the emergence of treatment resistance. Clearly, further investigations are strongly encouraged to use ctDNA as a biological material to elucidate the underlying molecular mechanisms that sustain resistance to the applied treatment and, on this basis, to objectively propose complementary lines of personalized therapy.

Finally, prospective trials using ctDNA testing to detect cancer early have shown promise, though sensitivity for early-stage disease remains limited [35]. For example, Wang and colleagues developed a highly sensitive method for detecting mutations in ctDNA through a well-designed case–control study [36]. The ARIC study is exemplary because it includes 15,792 participants aged 45–64 years. Among those participants with continuous blood collection and no cancer history, investigators detected ctDNA within three years before a cancer diagnosis, indicating the potential of ctDNA follow-up to identify cancer with a significant lead time.

To conclude at this stage, ctDNA measurement is already clinically established for domains like selected actionable mutations in advanced NSCLC. In contrast, some areas still require stronger prospective validation, such as MRD-driven escalation/de-escalation and multicancer early detection.

## 4. ctDNA: Limits and Perspectives

Undoubtedly, ctDNA analyses can now routinely and reliably detect mutations, such as EGFR mutations, in the management of lung cancer, thereby guiding targeted therapy [37]. However, it must be emphasized that tissue sampling remains recommended for exploring targetable tumor mutations in most solid tumors [8], particularly if the ctDNA result is negative. Thus, ctDNA analyses have not yet taken a central role in routine targeted therapy based on individual genomic profiling. Nevertheless, some optimism is justified, as ctDNA analyses can provide valuable information for disease monitoring and clinical decision-making. In this stage of MRD detection, a tumor-informed strategy for ctDNA genomic analysis may logically offer higher precision than a tumor-agnostic approach, as clearly demonstrated in colorectal cancer in a direct comparison [38]. However, a key challenge remains in determining the optimal pathways for integrating ctDNA status into clinical practice. Several options are available, with an essential aspect being the analytical conditions, from blood sampling to the final ctDNA analysis. Blood sampling conditions, including the time required for preparation, are critical, as platelet DNA release can influence the total ctDNA quantity in the sample and significantly impact the sensitivity of the analysis [39]. Another obstacle to routine adoption of ctDNA analyses is the variability in the measurement units used for ctDNA quantification (variant allele frequency, copy number alteration, DNA methylation, ppm), as underlined by Yin and coworkers [40]. Such variability complicates the generalization of clinical conclusions and underscores the need to establish standardized ctDNA concentration thresholds.

Recent advances in artificial intelligence have the capacity to increase the clinical impact of ctDNA in cancer management. Emerging computational strategies support this perspective [41]. For example, incorporating longitudinal variables greatly improves predictive accuracy compared to static models [38,42], demonstrating the potential for adaptive, ctDNA-informed therapeutic strategies. Complementary translational insights have highlighted the importance of integrating computational frameworks that bridge molecular dynamics with clinical outcomes [43]. With the help of advanced computational modeling, ctDNA could become a key tool for dynamic and personalized cancer care, provided methodological rigor and clinical validation are prioritized. A prime example of this methodological rigor is the recent development by Zhu and co-workers of a deep-learning model called “Fragle,” which accurately quantifies ctDNA using low-pass whole-genome sequencing across multiple cancer types and control groups [44]. The “Fragle model” estimates ctDNA fractions directly from genome-wide ctDNA fragment length distributions, capturing subtle fragmentation patterns linked to tumor-derived DNA. The authors explored the capacity of the Fragle model to detect MRD. Thus, in a cohort of patients with lung cancer, ctDNA levels estimated by Fragle were able to risk-stratify patients who were classified as ctDNA-negative by applying a commercial tumor-agnostic targeted sequencing assay. Interestingly, this fragmentomics approach does not require tumor-informed mutation profiling, thereby broadening the scope of liquid biopsy applications across different tumor types and clinical settings. Conversely, Costa and colleagues reported that AI can improve ctDNA testing for early lung cancer detection in healthy individuals and at-risk groups [43]. These advances suggest that ctDNA may progressively evolve into a real-time molecular sensor embedded within predictive clinical frameworks.

Such integration holds significant clinical promises by enabling adaptive treatment allocation, rational escalation or de-escalation of therapy, and facilitating biomarker-driven trial designs. Moreover, as Landon emphasizes [6], widespread adoption of these approaches depends on rigorous prospective validation, harmonized assays, reproducibility across platforms, and, ultimately, regulatory standardization. The development of AI-based algorithms, especially for ctDNA assays, must undergo rigorous validation to ensure mathematical and algorithmic rigor, supported by well-designed clinical trials. Given the current state of evidence, routine clinical use of ctDNA in cancer management has not yet been achieved, but with continued validation and standardization, it has the potential to revolutionize patient care. Future research directions could include large-scale validation of emerging AI algorithms and analytical platforms to assess cross-platform reproducibility and validate their mathematical and algorithmic performance in prospective clinical studies.

## 5. Conclusions

ctDNA measurement has a high clinical potential to identify actionable mutations for targeted therapy. The strong prognostic value of ctDNA testing has been clinically demonstrated, as has its capacity to predict treatment response. Various aspects related to the usability and clinical applicability of routine ctDNA measurement remain to be addressed before its implementation in clinical practice.

## Figures and Tables

**Table 1:** ctDNA, tumor-informed vs. tumor-agnostic.

Characteristics	Tumor-Informed	Tumor-Agnostic
Sequencing of the patient’s tumor	Yes	No
Plasma investigations	Specific mutations already in the patient tumors	Standard panel of mutations commercial kit
Sensitivity for minimal residual disease (MDR)	Very elevated	Relatively low
Risk for false positive	Low	More elevated
Duration of analysis	Long	Rapid
Necessity for a tumoral sample	Yes	No
Analytical cost	Relatively high	Simpler to apply

## Data Availability

Not applicable. There is no data or materials involved in this review.

## Abbreviations

The following abbreviations are used in this manuscript:

ctDNA	circulating tumor DNA
ddPCR	droplet digital polymerase chain reaction
ESMO	European Society for Medical Oncology
FDA	Food and Drug Administration
MRD	minimal residual disease
NGS	next-generation sequencing
WGS	whole-genome sequencing
